# Edge disturbance drives liana abundance increase and alteration of liana–host tree interactions in tropical forest fragments

**DOI:** 10.1002/ece3.3959

**Published:** 2018-04-02

**Authors:** Mason J. Campbell, Will Edwards, Ainhoa Magrach, Mohammed Alamgir, Gabriel Porolak, D. Mohandass, William F. Laurance

**Affiliations:** ^1^ Centre for Tropical Environmental and Sustainability Science (TESS) College of Science and Engineering James Cook University Cairns Queensland Australia; ^2^ Basque Centre for Climate Change‐BC3 Leioa Spain; ^3^ Estación Biológica de Doñana (EBD‐CSIC) Sevilla Spain; ^4^ Root and Soil Biology Lab Department of Botany Bharathiar University Coimbatore India

**Keywords:** climbing guild, competition, disturbance, fragmentation, infestation, management, vine

## Abstract

Closed‐canopy forests are being rapidly fragmented across much of the tropical world. Determining the impacts of fragmentation on ecological processes enables better forest management and improves species‐conservation outcomes. Lianas are an integral part of tropical forests but can have detrimental and potentially complex interactions with their host trees. These effects can include reduced tree growth and fecundity, elevated tree mortality, alterations in tree‐species composition, degradation of forest succession, and a substantial decline in forest carbon storage. We examined the individual impacts of fragmentation and edge effects (0–100‐m transect from edge to forest interior) on the liana community and liana–host tree interactions in rainforests of the Atherton Tableland in north Queensland, Australia. We compared the liana and tree community, the traits of liana‐infested trees, and determinants of the rates of tree infestation within five forest fragments (23–58 ha in area) and five nearby intact‐forest sites. Fragmented forests experienced considerable disturbance‐induced degradation at their edges, resulting in a significant increase in liana abundance. This effect penetrated to significantly greater depths in forest fragments than in intact forests. The composition of the liana community in terms of climbing guilds was significantly different between fragmented and intact forests, likely because forest edges had more small‐sized trees favoring particular liana guilds which preferentially use these for climbing trellises. Sites that had higher liana abundances also exhibited higher infestation rates of trees, as did sites with the largest lianas. However, large lianas were associated with low‐disturbance forest sites. Our study shows that edge disturbance of forest fragments significantly altered the abundance and community composition of lianas and their ecological relationships with trees, with liana impacts on trees being elevated in fragments relative to intact forests. Consequently, effective control of lianas in forest fragments requires management practices which directly focus on minimizing forest edge disturbance.

## 1 INTRODUCTION

Habitat fragmentation is globally ubiquitous (Bhagwat, 2014; Riitters, Wickham, Costanza, & Vogt, 2016; Wade, Riitters, Wickham, & Jones, 2003). In fact, it is currently estimated that 70% of the world's remaining forest is within 1 km from a forest edge (Haddad et al., 2015). This is important as the fragmentation of forests and associated edge effects can reduce biodiversity and degrade forest functioning (e.g., Fahrig, 2003; Laurance, Delamonica, Laurance, Vasconcelos, & Lovejoy, 2000; Laurance et al., 2002, 2011; Magrach, Laurance, Larrinaga, & Santamaria, 2014a; Saunders, Hobbs, & Margules, 1991). For instance, forest fragments (32 m^2^–100 ha) are estimated to possess 13%–75% less diversity than comparable nonfragmented forests (Haddad et al., 2015) with the majority of the lost diversity often the most iconic components, such as big trees and large mammals (Chiarello, 1999; Gibson et al., 2013; Laurance, 1997b; Laurance et al., 2000; Oliveira, Santos, & Tabarelli, 2008). In addition, forest fragmentation is also known to alter or degrade many beneficial ecological processes such as pollination and seed dispersal (Campbell, Laurance, & Magrach, 2015a; Campbell, Magrach, & Laurance, 2015b; Laurance et al., 2002; Magrach et al., 2014a; Peh, Lin, Luke, Foster, & Turner, 2014; Terborgh et al., 2001).

In the tropics, large‐scale deforestation has resulted in forest fragments now representing a substantial proportion of the remaining forested area in many regions such as the Atlantic forest of Brazil, West Africa, and the Atherton Tableland of northeastern Australia (Ouedraogo et al., 2011; Ribeiro, Metzger, Martensen, Ponzoni, & Hirota, 2009; Winter, Bell, & Pahl, 1987). In such regions, forest fragments provide the primary or sole repository for the preservation of many rare and endangered species and threatened ecosystems (Arroyo‐Rodriguez & Mandujano, 2006; Arroyo‐Rodriguez, Pineda, Escobar, & Benitez‐Malvido, 2009; Guindon, 1996). Maximizing the conservation value of forest fragments requires that fragments are not only retained, but are managed effectively, which necessitates an understanding of their internal ecology.

One of the major ecological interactions altered by fragmentation is the relationship between trees and lianas. Lianas detrimentally impact trees by limiting seedling recruitment (Schnitzer & Carson, 2010; Schnitzer, Dalling, & Carson, 2000), damaging saplings and decreasing tree growth and fecundity (Stevens, 1987), competing with trees for limited resources (Pasquini, Wright, & Santiago, 2015; Reid, Schnitzer, & Powers, 2015; Rodríguez‐Ronderos, Bohrer, Sanchez‐Azofeifa, Powers, & Schnitzer, 2016; Schnitzer, Kuzee, & Bongers, 2005), and increasing tree mortality (Ingwell, Wright, Becklund, Hubbell, & Schnitzer, 2010). In addition, lianas can modify the functioning of a forest by reducing carbon storage capacity (Durán & Gianoli, 2013; van der Heijden, Schnitzer, Powers, & Phillips, 2013; Schnitzer, van der Heijden, Mascaro, & Carson, 2014), re‐distributing nutrients (Kazda, 2015; Powers, Kalicin, & Newman, 2004; Schnitzer & Bongers, 2011), altering tree‐species composition (Clark & Clark, 1990; Laurance et al., 2001; Schnitzer & Bongers, 2002), threatening epiphytic ferns (Magrach, Rodríguez‐Pérez, Campbell, & Laurance, 2014b), and limiting or changing the trajectory of tree‐species succession within treefall gaps (Schnitzer & Bongers, 2005; Schnitzer & Carson, 2001, 2010; Schnitzer et al., 2000). Thus, lianas can have significant impacts on both the biota and functioning of remnant forest fragments. Understanding the ecological interactions between lianas and their host trees is critical for successfully managing remnant forest fragments, especially those with high conservation value.

There is strong support for the observation that lianas preferentially impact certain ecological “guilds” of tree species such as late‐successional/climax species (Campbell et al., 2015a, 2015b; Clark & Clark, 1990; Laurance et al., 2001; Schnitzer et al., 2000), although there is little evidence that this occurs at a species‐specific level (Garrido‐Perez & Burnham, 2010; Hegarty, 1991; Pérez‐Salicrup, Sork, & Putz, 2001). The enhanced liana infestation rates on late‐successional tree species is likely due to the advanced age (and thus time available for possible infestation) of these trees and certain character traits they possess (Hegarty, 1991; Schnitzer & Bongers, 2002). Such traits include bark morphology and chemical composition (Boom & Mori, 1982; Carsten, Juola, Male, & Cherry, 2002; van der Heijden, Healey, & Phillips, 2008; Putz, 1980; Talley, Setzer, & Jackes, 1996), buttresses (Black & Harper, 1979; Boom & Mori, 1982; Putz, 1980), leaf shedding and leaf and stem flexibility (Maier, 1982; Putz, 1984a; Rich, Lum, Munoz, & Quesada, 1987), tree/trellis diameter (Clark & Clark, 1990; Perez‐Salicrup & de Meijere, 2005; Pérez‐Salicrup et al., 2001; Putz, 1984b), spines (Maier, 1982; Putz, 1984a; Rich et al., 1987), liana–host distance and availability (Arroyo‐Rodriguez & Toledo‐Aceves, 2009; Campbell et al., 2017; Muthuramkumar et al., 2006; Roeder, Slik, Harrison, Paudel, & Tomlinson, 2015), and synergisms among these traits (Sfair, Rochelle, Rezende, & Martins, 2016). As such, a comparative assessment of the predominant tree traits between intact and fragmented forests, and their association with liana infestation, may be of use as a proxy to determine how forest fragmentation impacts liana–tree interactions and contributes to increased liana abundance within fragmented forests (Laurance et al., 2001).

The total abundance of lianas is known to be positively associated with forest edges and areas of disturbance (Laurance et al., 2001, 2014b; Ledo & Schnitzer, 2014; Magrach et al., 2014b; Mohandass, Campbell, Hughes, Mammides, & Davidar, 2017; Mohandass, Hughes, Campbell, & Davidar, 2014; Putz, 1984b). High liana abundances at forest edges are likely due to edge effects (e.g., Harper et al., 2005; Laurance et al., 2002; Magnago et al., 2016; Murcia, 1995; Williams‐Linera, 1990), in particular to the increased availability of climbing trellises (i.e., smaller‐stemmed trees; Balfour & Bond, 1993; Chittibabu & Parthasarathy, 2001; Londre & Schnitzer, 2006; Putz, 1984b; Williams‐Linera, 1990). Moreover, forest edges are often more disturbed than forest interiors (Laurance et al., 1997, 2011, 2018; Magnago, Rocha, Meyer, Martins, & Meira‐Neto, 2015), resulting in increased desiccation and light levels. These conditions preferentially favor lianas over trees, through mechanisms such as differential recruitment success and resource‐interception capacity (Andrade, Meinzer, Goldstein, & Schnitzer, 2005; Chen et al., 2015; Ledo & Schnitzer, 2014; Oliveira, deMello, & Scolforo, 1997; Perez‐Salicrup & Barker, 2000; Rodríguez‐Ronderos et al., 2016; Schnitzer & Carson, 2010). Consequently, it is important that any study of liana–tree interactions examine the spatial distribution of lianas in relation to forest edges.

Analyzing the abundance of lianas within climbing guilds between intact and fragmented forests can also be used to assess liana–host tree interactions. For example, assessing the proportion of lianas within climbing guilds can reveal the current trellis availability and thus the successional state of the forest (Hegarty & Caballe, 1991; Laurance et al., 2001; Mohandass et al., 2014; Putz, 1984b). This is possible because lianas within different climbing guilds utilize trellises of differing maximal diameter (Balfour & Bond, 1993; Putz, 1984b, 1990; Putz & Chai, 1987). For instance, climbers that attach with adhesive roots are not limited by trellis (i.e., tree branch or trunk) size, whereas mainstem twining and branch climbers use larger trellises (branches) than do tendril and hook climbers (Balfour & Bond, 1993; Putz, 1984b, 1990; Putz & Chai, 1987).

Here, we compare the response of lianas to forest fragmentation, edge effects, and liana–host tree interactions in fragmented and intact forests, within the heavily fragmented landscape of the Atherton Tableland in northeastern Australia. In this study, we aimed to determine whether forest fragmentation alters the liana community on forest edges and if so, whether this is predominantly driven by landscape level fragmentation impacts or those at a smaller habitat (i.e., within patch) spatial scale. To determine (i) the separate influence of fragmentation and edge effects on liana abundance, tree infestation rates, and liana size (diameter at breast height [DBH]), we asked: what were the important environmental and ecological predictors associated with these measures at the landscape level (in fragmented and intact forests) and are these similar? We hypothesized that liana abundance and tree infestation rates would be greater on fragmented forest edges given the higher rates of disturbance they are known to experience (Laurance et al., 2018) as disturbance is a primary driver of liana abundance (Schnitzer & Bongers, 2002). Second, we assessed habitat scale traits by asking: (ii) do tree morphological traits (tree bark type or buttressing) and tree location, with respect to the forest edge, influence liana infestation rates within fragmented and intact forests and if so are these influences similar between these two forest types? We hypothesized that liana abundance and tree infestation rates would be greater on smaller‐sized trees and those with rough bark given that these traits can facilitate colonization by lianas. Again, we also hypothesized that trees on fragmented forest edges would experience greater levels of infestation than those of intact forests given the increased disturbance in those locations (and thus small tree/trellis size) and that these variables would have a negative relationship with distance to forest edge. Finally, to determine the response of the liana community to forest fragmentation and edge effects, we asked: (iii) does the liana community climbing‐guild composition vary by forest type (fragmented or intact) and is this relationship affected by the distance to the forest edge? We hypothesized that liana guilds utilizing smaller‐sized trellises would increase disproportionately (when compared to other climbing guilds) in fragmented forests and closer to forest edges due to the increased availability of smaller trellises at these locations.

## 2 MATERIALS AND METHODS

### 2.1 Study area

**Figure 1:**
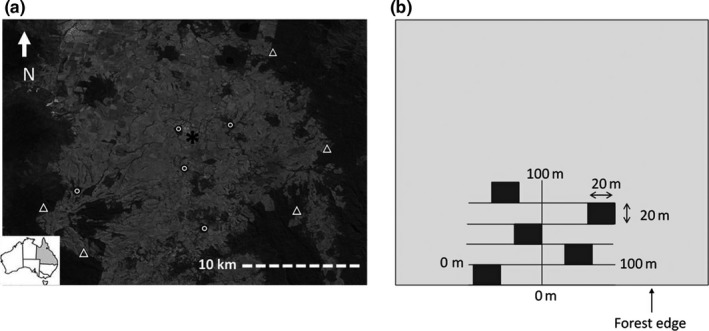
(a) Location of the ten study sites on the Atherton Tablelands, Australia. Study sites are indicated as triangles for intact forests and circles for fragmented forest. Malanda as the nearest town is indicated with an asterisk; (b) the design of vegetation sampling at each study site wherein five 20 × 20 m plots were stratified and randomly placed with respect to the forest edge

Our study was located on the Atherton Tableland, northeastern Queensland, Australia (Figure 1a). The Atherton Tableland is an upland, hilly plateau ranging in elevation from ~600 to 1,100 m. Mean annual precipitation of the Atherton Tablelands ranges from 1,400 to 3,000 mm due to a localized northwest (low) to southeast (high) rainfall gradient; however, the variation in the study area is much less (~200 mm). Most annual rainfall occurs during a pronounced wet season from January to April. The area is also prone to cyclonic episodes during the wet season (Turton, 2012) which can result in increased precipitation and forest disturbance (Turton & Siegenthaler, 2004; Turton & Stork, 2009).

The local vegetation of the study area is remnant fragments and regrowth of a larger rain forest expanse that previously covered the Atherton Tableland, now isolated by a predominantly agricultural land‐use matrix (Figure 1a). Deforestation of this area has been extensive with over 76,000 ha cleared for cattle pasture and crop lands (Winter et al., 1987). Additionally, most of the remnant rain forest vegetation has been selectively logged for valuable hardwood timber species such as Red Cedar (*Toona ciliata*) (Eacham Historical Society, 1979, 1995; Pearson, 2008). Nevertheless, many of these forest fragments form a large part of the greater Wet Tropics World Heritage area (UNESCO, 1988).

The remnant vegetation of the area is described as complex mesophyll vine forest and notophyll vine forest with drier areas transitioning into complex semievergreen notophyll vine forest (Queensland Herbarium, 2015; Tracey, 1982). Within the complex mesophyll vine forest, multiple intact canopies may be present with the upper canopy averaging a height of 20–40 m and emergent trees reaching 55 m (Queensland Herbarium, 2015; Tracey, 1982). Deciduous tree species are rare; however, woody lianas, epiphytes, and ferns are common resulting in a complex forest structure (Tracey, 1982).

Volcanic soils, namely krasnozems, occur on the level to undulating plains and rise in the study region while steeper mountainous areas generally comprise nutrient‐poor granite and rhyolite‐derived soils (Malcom, Nagel, Sinclair, & Heiner, 1999).

### 2.2 Study sites and sampling design

Ten sites were selected for study, comprising five forest fragments and five sites in nearby intact rain forest (Figure 1a). Forest fragments were selected to: minimize variation in total area (23–58 ha), and thus limit patch‐area effects on liana abundance (Laurance et al., 2001; Mohandass et al., 2014), comprise remnant forest of similar successionary status (selected using vegetation data provided by the Wet Tropics Management Authority (WTMA), Cairns, Australia (WTMA, 2009), the managing body for the world heritage area), and to ensure that they were all of a similar age (created prior to 1950) and surrounding matrix type (surrounded by cattle pastures) to lessen possible confounding effects of fragment age or surrounding matrix type. Intact‐forest sites were selected to be as spatially close as possible to the fragments, with the largest between‐site distance for all sites being <23 km and the smallest fragment to intact site distance 3.2 km. Intersite distance was minimized to lessen variation in environmental variables known to influence liana abundance; in particular rainfall, elevation, and soil type (DeWalt et al., 2010, 2015; Laurance et al., 2001; Schnitzer, 2005; Schnitzer & Bongers, 2002). The intact forest sites were also intact, remnant forest of similar successionary status to the fragments (again selected using the WTMA vegetation data). Finally, both fragments and intact forest sites were selected to ensure they were overlying volcanic soils (krasnozems) to limit confounding effects of differing soil types.

At each site, we used a linear transect to establish five 20 × 20 m plots stratified at five distance classes perpendicular to the forest edge (0–20, 20–40, 40–60, 60–80, and 80–100 m; Figure 1b) for a total (*n*) of 50 plots. At each 20 m distance into the forest, plots were randomly located along a 100‐m‐long transverse transect (Figure 1b) to increase their statistical independence. The smallest distance between plots at any site was 20 m, and all plots were greater than 100 m from any other forest edge to avoid confounding influences of multiple forest edges.

### 2.3 Liana measures

From March 2012 to February 2014, liana abundance, DBH, and climbing guild were determined for each liana within all individual plots at each of the 10 sites. Liana abundance was determined by counting all liana stems ≥1 cm DBH within each plot. Unless clearly joined, stems were assumed to be individual lianas with no excavation conducted to determine below ground connections. The location for DBH measurement of each liana stem was determined by liana growth morphology as per current methodology (Gerwing et al., 2006; Schnitzer, DeWalt, & Chave, 2006; Schnitzer, Rutishauser, & Aguilar, 2008), and plot‐level comparisons were made using median liana size per plot. Additionally, each liana was assigned to one of five climbing guilds: mainstem twiner, branch twiner, tendril climber, root climber, and scrambler (Putz, 1984b) and trees (≥10 cm DBH) used as climbing supports were identified and given a unique tag number.

### 2.4 Environmental and structural parameters of fragmented and intact forests

To characterize the environmental and ecological conditions of fragmented and intact forest sites, we examined physical and structural parameters of forests which are known to influence liana abundance as identified using the liana literature and discussed in the following paragraphs.

To assess forest disturbance, two measures were examined for each plot: canopy cover and the number of fallen trees (≥10 cm diameter). Canopy cover was estimated at the four corners and the center of each plot and was measured by averaging four spherical densiometer readings taken facing the cardinal directions (N, E, S, W) at each point.

To determine physical traits of plots, we examined their slope and elevation. The degree of slope of each plot was calculated using a clinometer, while elevation of all sites was assessed using climatic model interpolations data provided by WTMA (WTMA, 2009). These data were also assessed to determine the annual rainfall (mm) and dry quarter rainfall (July–September, mm) of sites.

The structural parameters of fragmented and intact forest sites were examined through assessment of the resident rattan (*Calamus* spp.) population, tree population and plot live carbon storage assessment. Relative rattan abundance was recorded for each plot by averaging the counts of independent rattan stems along four, 3‐m longline intercept transects located in each corner of the examined plots (for detailed methods see Campbell et al., 2017).

The tree population was assessed by counting all trees (≥10 cm DBH) within each plot and measuring their DBH at 1.3 m height or above any buttresses. Trees were also scored into bark type categories of “smooth,” “rough,” or “shedding” and buttress categories of “present” or “absent.” These classifications were visually determined by the same researcher throughout the study (MJC).

Relative live plot carbon storage was estimated by combining carbon above ground estimates of all live trees ≥10 cm and lianas ≥1 cm within a plot. Liana biomass was calculated using the liana‐specific allometric equation (Equation (1)) developed by Schnitzer et al. (2006):

(1) AGB=exp[−1.484+2.657ln(D)]

In this model, *D* is the diameter at 130 cm from the roots (with the location determined as per Gerwing et al. (2006)) expressed in centimeters, while AGB is the predicted above ground oven‐dry weight of the liana in kilograms.

Tree above ground biomass (ABG) was calculated using the allometric equation (Equation (2)) developed by Chave et al. (2005) (see below) as Preece, Crowley, Lawes, and van Oosterzee (2012) compared the accuracy of multiple biomass estimation methods for forests within the Wet Tropics bioregion (within which the study area is found) and concluded that the Chave et al. (2005) allometric provided the best and most reliable estimate for the region. To convert AGB into biomass carbon storage, we used a conversion factor of 0.47 which is the recommended value from the Intergovernmental Panel for Climate Change for tropical forests (IPCC, 2006). In addition, relative AGB was calculated using a single wood density estimate at the reported default value for Australian tropical forests of 0.5 g/cm^3^ (500 kg/m^3^) (Department of Climate Change and Energy Department of Climate Change and Energy Efficiency 2010). Consequently, relative tree AGB estimates were calculated using the following equation:

(2) $$\begin{array}{cc} \text{AGB}\;=\; & \rho^{*}{\exp\;(-1.499+2.148\ln\;\left(dbh\right)+0.207\;\left(\ln\;\left(dbh\right)\right)}^{2}\\ & -0.0281\;{\left(\ln\;\left(dbh\right)\right)}^{3}) \end{array}$$

where AGB is measured in kg, dbh is measured in cm, and ρ is wood density measured in g/cm^3^.

Relative above ground biomass estimates for both lianas and trees were then converted to relative carbon estimates (Equation (3)) using the formula:

(3) Carbon=AGB∗0.47

### 2.5 Data analysis

#### 2.5.1 Environmental and structural parameters of fragmented and intact forests

Disturbance and forest gap dynamics along with the availability and size of trees (liana supports) are known to be the major drivers of the distribution of lianas within forests (Ledo & Schnitzer, 2014; Schnitzer & Bongers, 2005; Schnitzer & Carson, 2010; Schnitzer et al., 2000). To assess these traits within fragmented and intact forests, canopy cover, tree abundance, and tree DBH were compared along with their relationships with the previously identified (see above) environmental and structural parameters (other than tree bark type and buttressing which, due to sample size limitations, were assessed in log‐linear models below). The relationship between these response variables and the environmental and structural parameters was compared using individual generalized linear mixed models (GLMMs) in the glmmADMB (Fournier et al., 2012) and lme4 (Bates, Maechler, Bolker, & Walker, 2015) packages. GLMMs were selected to analyze the data given that multiple explanatory factors simultaneously influenced the response variables, the response variables were nonnormally distributed, and the sample units (plots) were nested (by site).

Prior to model generation, we checked for correlated predictor variables following the protocol of Zuur, Ieno, and Elphick (2010). One variable was subsequently removed: the mean dry quarter rainfall. To prevent undue influence of any explanatory variable due to unit of measurement, all explanatory variables used in the model were standardized ((*x* − mean(*x*))/*SD*(*x*)). Standardizing in this manner has the additional benefit that the effects sizes of all variables included in the model can be directly compared via model coefficients. Additionally, as there were five plots within each site (stratified by forest edge distance), plots were not fully independent. As such, we included site ID as a random effect. Consequently, in each model‐fitting exercise we selected a priori a global model in which the response variable (tree abundance, tree DBH, and canopy cover) was examined as a function of the following variables (with the response variable removed from this list in their respective GLMM): the number of fallen logs (≥10 cm diameter), plot elevation (m), plot slope (degrees), mean annual rainfall (mm), plot distance to forest edge (m), mean tree abundance, tree DBH (cm), and plot carbon storage (tonnes/ha), relative rattan abundance, liana abundance, liana DBH and proportionate liana infestation of trees, canopy cover (%), tree abundance, tree DBH (cm), and the interaction between the forest type and edge distance. The most parsimonious models were then determined using backwards, stepwise regression with selection based on lowest AIC model values using the drop1 function of Program R (R Core Team, 2015). The most parsimonious model was defined as that which included the minimum number of terms to produce the best possible explanation of the response variable (lowest AIC value), and may or may not have contained traditionally significant (*p* < .05) variables. Tree abundance was examined using a poisson GLMM, and tree DBH and canopy cover were examined using individual gamma GLMMs with log link. Canopy cover was also logit‐transformed prior to model initiation.

#### 2.5.2 The influence of fragmentation on liana infestation of trees, liana abundance, and liana DBH

Once we had quantified the variation in canopy cover, tree abundance and tree DBH between fragmented and intact forests and their interactions with the environmental and structural parameters, we then construct individual GLMMs to identify the influence of fragmentation on (i) the proportion of trees infested by lianas per plot, (ii) liana abundance per plot, and (iii) liana size (DBH). All model construction and fitting was performed as per the previous methods (see above). The proportion of trees infested by lianas was examined using a binomial GLMM with a logit link, liana abundance using a negative binomial GLMM, and the liana DBH examined using a gamma GLMM with log link. Furthermore, where examination of the residuals from the final model revealed incorrect model fit, model fit was further improved by including a quadratic term. This occurred after checking residual diagnostics for models describing the proportion of trees infested by lianas and liana abundance, with curvature in both cases related to distance to the forest edge (see Section 2.1).

#### 2.5.3 Host‐tree morphology and forest effects

A log‐linear model (Poisson with log link) was used to determine the relationship between host‐tree morphological traits and the impact of forest effects. These were assessed by examining the relationship between the categorical variables of tree buttress presence (yes or no), tree bark type (smooth, rough, or shedding), forest type (fragmented or intact), distance to the forest edge (0–20, 20–40, 40–60, 60–80, and 80–100 m), and whether a tree was infested by one or more lianas (yes or no).

#### 2.5.4 Infesting liana climbing guilds, forest type, and environmental traits

To determine the relationship between infesting liana traits and the impact of forest effects, we used a log‐linear model as in the tree‐host traits model above. We compared the categorical variables: liana climbing guild type (branch climber, hook climber, mainstem twiner, root climber, scrambler, tendril climber, unknown), forest type (fragmented or intact), distance to the forest edge (0–20, 20–40, 40–60, 60–80, and 80–100 m), and whether a tree was infested by lianas (yes or no). All analyses were performed in Program R (R Core Team, 2015).

## 3 RESULTS

### 3.1 Environmental and structural parameters of fragmented and intact forests

Tree abundance was significantly lower in fragmented forests than in intact forests but was higher on forest edges than on forest interiors (see Table S1). As expected, tree abundance was significantly and positively related to relative forest carbon; however, it was significantly and negatively related to altitude (Table S1).

Tree size (DBH) was significantly higher in fragmented forests than in intact forests and was also higher in sites with greater canopy cover, at higher altitude, where large lianas were present and sites with greater relative forest carbon (see Table S2).

Canopy cover was significantly lower in fragmented that in intact forests and was lower on forest edges than on forest interiors (see Table S3). The reduction in canopy cover also penetrated significantly further into the edges of fragmented than intact forests (Table S3). Canopy cover was also found to be significantly and negatively related to altitude (Table S3).

### 3.2 Environmental and structural predictors of tree infestation by lianas

**Figure 2:**
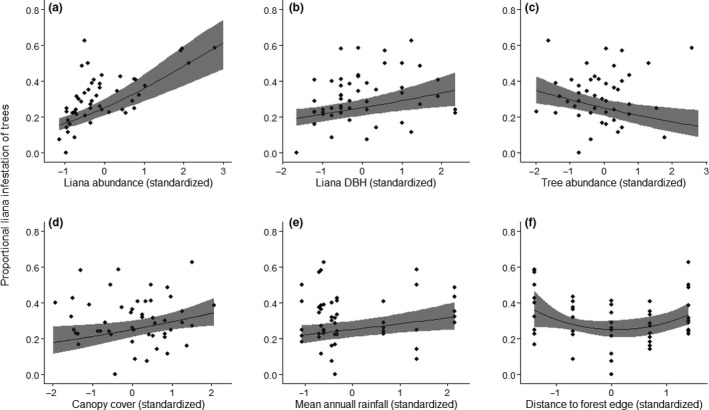
The relationship between proportional tree infestation by lianas and (a) liana abundance, (b) liana DBH (median per plot), (c) tree abundance, (d) canopy cover, (e) mean annual rainfall, and (f) midplot distance to the forest edge. The trend lines are predicted values, and shaded areas represent the 95% confidence intervals

Tree infestation by lianas was not significantly related to forest type (fragmented or intact) (Table 1) with an average of ~29% (*SE* ±0.024) of trees infested in fragments and ~32% (*SE* ±0.029) in intact forest. Tree infestation by lianas was significantly and positively related to increasing liana abundance, liana DBH, canopy cover, and mean annual rainfall (Table 1; Figure 2). Of these parameters, liana abundance had the greatest influence on the proportional liana infestation of trees with the highest relative effect size of 0.517 (*SE* ±0.079) (Table 1). Tree infestation by lianas significantly decreased with increasing tree abundance but was parabolically related to the forest edge distance with more trees infested by lianas on forest edges and in forest‐interior plots and fewer in those plots in between (Table 1; Figure 2).

**Table 1:** The most parsimonious generalized linear mixed model (binomial) for the influence of forest fragmentation effects and environmental and forest structural parameters on proportional tree infestation by lianas

	Estimate	*SE*	*Z* value	*p*
Intercept	−1.086	0.122	−8.881	**<.001**
Forest edge distance	−0.040	0.107	−0.379	.704
Quadratic term forest edge distance (*x*_1_ + $x_{1}^{2}$)	0.234	0.102	2.286	**.022**
Liana abundance	0.517	0.079	6.481	**<.001**
Tree abundance	−0.232	0.083	−2.798	**.005**
Liana DBH (median per plot)	0.202	0.064	3.114	**.001**
Canopy cover	0.216	0.091	2.364	**.018**
Mean annual rainfall	0.161	0.063	2.528	**.011**

Forest edge distance = middistance of plot to the forest edge (m) and this was analyzed using a quadratic term based on initial residual diagnostics. All explanatory variables were standardized prior to the analysis ((*x* − mean(*x*))/*SD*(*x*)).

### 3.3 Environmental and structural predictors of liana abundance

**Figure 3:**
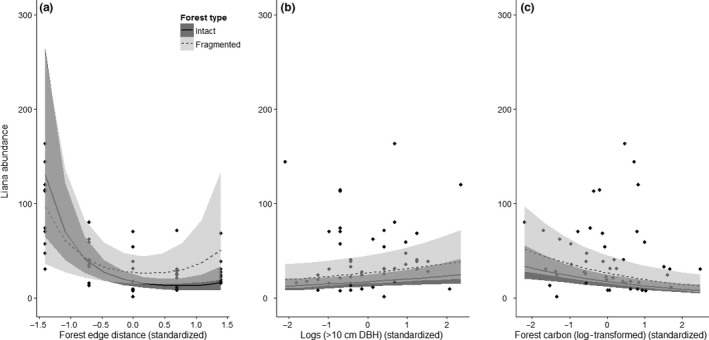
The relationship between liana abundance and the interaction of forest type and (a) distance to the nearest forest edge, (b) fallen logs, and (c) stored forest carbon (log10‐transformed). The individual trend lines are predicted values and show the significant interaction forest type and forest edge distance. Shaded areas represent the 95% confidence intervals

At the landscape level, we recorded a total liana abundance of 2,124 (*n*) stems. Liana abundance was significantly and positively related to forest fragmentation and an increase in the number of fallen logs in a forest (Table 2, Figure 3). However, liana abundance significantly decreased with an increase in forest carbon storage (Table 2; Figure 3). Liana abundance was also significantly and parabolically related to forest edge distance with more lianas on forest edges and in forest‐interior plots and fewer in those plots in between (Table 2; Figure 3). Moreover, there was a significant interaction between forest type (fragmented or intact) and the distance to the nearest forest edge (Table 2; Figure 3). Of all parameters tested, forest‐edge distance had the largest influence on liana abundance with a relative effect size of −0.750 (*SE* ±0.162) (Table 2).

**Table 2:** The most parsimonious generalized linear mixed model (negative binomial) for the influence of forest fragmentation effects and environmental characteristics on liana abundance

	Estimate	*SE*	*Z* value	*p*
Intercept	2.839	0.186	15.25	**<.001**
Forest edge distance (m)	−0.750	0.162	−4.61	**<.001**
Quadratic term forest edge distance (*x*_1_ + $x_{1}^{2}$)	0.499	0.116	4.27	**<.001**
Forest type (Fragmented)	0.427	0.202	2.11	**.035**
Tree abundance	0.180	0.122	1.47	.140
Carbon	−0.307	0.083	−3.68	**<.001**
Altitude	0.156	0.092	1.70	.089
Fallen logs	0.156	0.078	2.01	**.044**
Canopy cover	0.246	0.142	1.73	.083
Forest edge distance:forest type interaction	0.520	0.164	3.16	**.001**

Forest edge distance = middistance of plot to the forest edge (m) and this was analyzed using a quadratic term based on initial residual diagnostics. All explanatory variables were standardized prior to the analysis ((*x* − mean(*x*))/*SD*(*x*)).

### 3.4 Environmental and structural predictors of liana DBH

**Figure 4:**
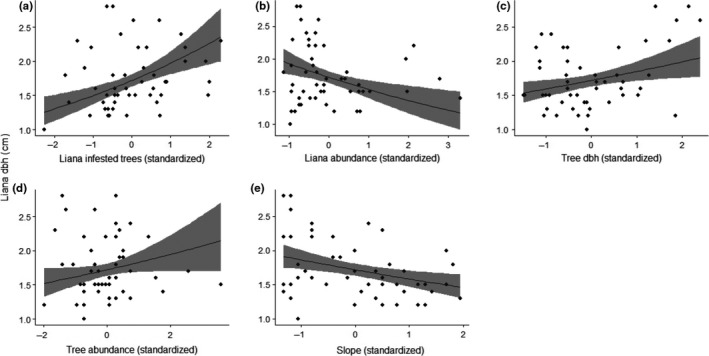
The relationship between liana diameter breast height (DBH, median per plot) and (a) proportion of trees infested by lianas, (b) liana abundance, (c) tree DBH, (d) tree abundance, and (e) slope. The trend lines are predicted values, and shaded areas represent the 95% confidence intervals

Liana DBH was significantly and positively related to both tree‐infestation rates and tree DBH, and there was a positive but nonsignificant relationship between liana DBH and tree abundance (Table 3; Figure 4). Conversely, liana DBH was negatively related to an increase in liana abundance and site slope (Table 3; Figure 4). Of the examined parameters, the number of liana‐infested trees had the largest positive influence on liana DBH with a relative effect size of 0.137 (*SE* ±0.034; Table 3). Conversely, liana abundance was the most negatively related parameter to liana DBH with a relative effect size of −0.115 (*SE* ±0.037) (Table 3).

**Table 3:** The most parsimonious generalized linear mixed model (gamma log link) for the influence of forest fragmentation effects and environmental characteristics on liana diameter breast height (median per plot)

	Estimate	*SE*	*t* value	*p*
Intercept	0.542	0.026	20.56	**<.001**
Proportionate liana infestation of trees	0.137	0.034	3.97	**<.001**
Liana abundance	−0.115	0.037	−3.11	**.001**
Tree diameter breast height (DBH)	0.073	0.028	2.55	**.010**
Tree abundance	0.061	0.032	1.92	.054
Slope	−0.081	0.027	−2.94	**.003**

Liana diameter breast height (cm) was measured as per current standard protocols (Gerwing et al., 2006; Schnitzer et al., 2006, 2008). All explanatory variables were standardized prior to the analysis ((*x* − mean(*x*))/*SD*(*x*)).

### 3.5 Host‐tree morphology and forest effects on liana‐infestation rates

The probability of a tree hosting a liana was primarily determined by its distance to the forest edge, with fragmentation status, tree bark type, or possession of buttresses having a limited affect (Table 4).

**Table 4:** The analysis of deviance for a log‐linear model investigating association between: trees infested with lianas (yes or no), forest type (fragmented or intact), distance to the forest edge (0–20, 20–40, 40–60, 60–80, and 80–100 m), buttress presence (yes or no), and bark type (smooth, rough, or shedding)

	*df*	Deviance	Residual *df*	Residual deviance	*p*
Null	NA	NA	119	3005.451	NA
Tree infested	1	220.284	118	2785.166	**<.001**
Forest type	1	17.823	117	2767.343	**<.001**
Edge	4	32.012	113	2735.331	**<.001**
Bark type	2	2549.900	110	184.913	**<.001**
Tree infested:edge	4	32.352	105	149.761	**<.001**
Forest:buttress	1	6.529	99	140.136	**.011**
Buttress:bark	2	11.811	81	111.681	**.003**
Forest:edge:buttress	4	9.627	68	84.437	**.047**
Tree infested:forest:buttress:bark	2	6.704	28	20.991	**.035**

*df*, degrees of freedom. Only significant findings are displayed.

### 3.6 Infesting liana climbing guilds, forest type, and environmental traits

Lianas that infested trees varied by both their distance to the forest edge and fragmentation status of the forest patch (Table 5). Moreover, there was a significant variation in the abundance of lianas within individual climbing guilds and differences between responses of different climbing guilds were associated with both the distance to the forest edge and forest fragmentation (Table 5).

**Table 5:** The analysis of deviance for a log‐linear model investigating association between: forest type (fragmented or intact), liana climbing guild (branch climber, hook climber, mainstem twiner, root climber, scrambler, tendril climber, unknown), distance to the forest edge (0–20, 20–40, 40–60, 60–80, and 80–100 m), whether the liana infested a tree (yes or no)

	*df*	Deviance	Residual *df*	Residual deviance	*p*
Null	NA	NA	139	3043.548	NA
Forest	1	12.064	138	3031.484	**<.001**
Guild	6	1032.740	132	1998.744	**<.001**
Edge	4	679.871	128	1318.874	**<.001**
Infesting liana	1	75.781	127	1243.092	**<.001**
Forest:guild	6	95.485	121	1147.607	**<.001**
Forest:edge	4	97.822	117	1049.785	**<.001**
Guild:edge	24	341.774	93	708.012	**<.001**
Forest:infesting liana	1	7.825	92	700.187	**.005**
Guild:infesting liana	6	211.509	86	488.678	**<.001**
Edge:infesting liana	4	14.513	82	474.165	**.006**
Forest:guild:edge	24	372.679	58	101.486	**<.001**
Forest:guild:infesting liana	6	22.505	52	78.981	**<.001**
Guild:edge:infesting liana	24	42.878	28	36.103	**.010**

*df*, degrees of freedom. Nonsignificant higher‐interaction terms were removed.

## 4 DISCUSSION

### 4.1 Liana abundance and habitat fragmentation

From our results, it is clear that forest edge disturbance and habitat fragmentation have significantly altered the liana community and the ecological relationship between lianas and trees within rainforests of the Atherton Tableland. We found forest fragmentation resulted in a significant increase in liana abundance. Furthermore, whereas liana abundance was significantly higher on the edges of both forest types, this effect penetrated further into the edges of fragmented than intact forests. It is likely that the increase in liana abundance at greater distances within fragmented forests is primarily due to increased disturbance on fragment edges. For example, canopy cover was significantly less within fragmented forests than in intact forests (Table S3). Furthermore, canopy cover decreased significantly in response to proximity to the forest edge in both forest types, but this occurred at a significantly greater rate in fragmented forests (Table S3). A decrease in canopy cover, which is found on forest edges or in treefall gaps, is well known to favor liana proliferation, often at the expense of tree recruitment, tree succession, tree growth, and forest carbon storage (Schnitzer & Carson, 2001, 2010; Schnitzer et al., 2000, 2014).

Liana abundance also significantly increased with increasing frequency of fallen logs (≥10 cm diameter) within a plot; an indicator of past forest disturbance (e.g., Attiwill, 1994). Moreover, liana abundance significantly decreased with increasing forest carbon storage, which is strongly positively associated with the presence of large trees (Slik et al., 2013) indicative of low rates of forest disturbance (Laurance, Ferreira, Rankin‐de Merona, & Laurance, 1998; Laurance et al., 2000, 2002, 2006a). Numerous studies have shown that fragment edges experience higher levels of disturbance than those of intact forests (e.g., Harper et al., 2005; Laurance et al., 2011, 2018; Saunders et al., 1991; Tabarelli, Lopes, & Peres, 2008) with others identifying localized forest disturbance as the primary driver of local liana abundance within a forest (Laurance et al., 2001; Ledo & Schnitzer, 2014; Schnitzer, 2015; Schnitzer & Bongers, 2011). Thus, our results of liana abundance increasing in fragments in response to disturbance are supported by previous findings of liana proliferation due to increase in forest disturbance (Ledo & Schnitzer, 2014).

### 4.2 Liana infestation of trees

The proportion of trees infested by lianas did not differ significantly between fragmented and intact forests. Nevertheless, liana abundance was a significant predictor of the infestation rates of trees. As distance to the forest edge strongly influences liana abundance, increased disturbance near the edges of forest fragments is not only driving differences in the spatial pattern of liana concentrations but also the probability that individual trees will be infested (Laurance, 1997a; Laurance et al., 2001). In fact, studies suggest that the mere proximity of lianas to potential host trees may be a primary determinant of host tree selection by lianas (Roeder et al., 2015) and thus an increase in local liana abundance (due to forest disturbance) would lead to an increase in local tree infestation probabilities.

The probability of trees infestation by lianas was also significantly influenced by the size of lianas (DBH), with a higher fraction of trees infested at sites with a larger median liana size than at sites with a smaller median liana size. The correlation between liana size and tree impact was previously noted by Putz who observed that there is a strong correlation between liana diameter and liana leaf area (Putz, 1983) and thus the effects of lianas on their supporting trees (Putz, 1984b). However, unlike liana abundance, median liana size within a fragment was positively related to decreased disturbance and the prevalence of mature forest traits (Hegarty & Caballe, 1991; Letcher, 2015). We found median liana size (DBH) to be positively and significantly related to factors associated with mature successional forest traits such as larger tree diameter, increasing canopy cover and decreasing tree abundance. Therefore, while sites with larger lianas (DBH) significantly contributed to tree infestation rates, their prevalence was significantly related to areas of forest with mature forest traits (Hegarty & Caballe, 1991; Letcher, 2015).

As both increased liana abundance and size (DBH) significantly contributed to liana infestation rates of trees within a forest, it is likely that patterns of disturbance and subsequent forest succession combine to determine liana infestation rates of trees within forest fragments. For example, initial forest disturbance can facilitate liana recruitment and abundance (Ledo & Schnitzer, 2014), with subsequent forest canopy closure in these areas resulting in lianas in the forest canopy (i.e., in general those ≥2 cm; Kurzel, Schnitzer, & Carson, 2006) being retained and increasing in size, but the canopy closure precluding additional liana stems successfully reaching the canopy (Letcher, 2015; Letcher & Chazdon, 2009; Putz, 1984b). Consequently, tree infestation and liana size distributions within forest fragments likely reflect forest dynamics and liana community age with distinct differences in community composition between larger lianas in older (less disturbed) areas and smaller lianas in younger forest sections (i.e., recently disturbed) (Letcher, 2015).

### 4.3 Infesting liana climbing guilds and host tree traits and their response to forest effects

Liana infestation of trees has previously been linked to the morphological and ecological traits of lianas themselves (e.g., the preferred size of climbing trellises used by different liana‐climbing guilds; Putz, 1984b; Putz & Chai, 1987). We found fragmentation of the rain forest significantly influenced liana infestation of trees, and these effects, in turn, resulted in substantial shifts in the relative abundance of liana climbing guilds. Proportions of total stems in different liana climbing guilds varied significantly in response to forest edge distance within and between both fragmented and intact forests. It is likely that the variation in liana guild composition between fragmented and intact forests can again be attributed to increased disturbance of fragmented forest edges (Laurance, 1997a; Laurance & Curran, 2008; Oliveira et al., 1997; Tabarelli et al., 2008). Disturbance is known to result in the proliferation of usually smaller successional trees and earlier successional forests (Chazdon, 2014; Laurance, 1997a, 2002; Laurance et al., 2002, 2006b; Tabarelli et al., 2008). These recruits increase the availability of smaller‐sized climbing trellises (i.e., small trees and branches), which are favored by tendril climbers and stem twiners which also proliferate there. Lianas that utilize larger climbing trellises (e.g., branch twiners) are more frequently found in mature forest (Putz, 1984b; Putz & Chai, 1987; Schnitzer & Bongers, 2002). Consequently, much of the changes in liana community composition and infestation rates in fragmented forests can be attributed to the effects of disturbance in determining the availability of different‐sized climbing trellises.

Morphological attributes of trees have also been suggested to influence the probability of liana infestation. For both, tree bark type (Boom & Mori, 1982; Putz, 1980) and buttress presence (Boom & Mori, 1982; Putz, 1980) have been noted as potential liana inhibitors. For instance, it has been suggested that flaky barked trees may shed lianas, while smooth bark trees may decrease the success of liana attachment (Putz, 1984b). Meanwhile, tree buttressing has been hypothesized to act as a mechanical barrier, preventing liana proximity and therefore attachment (Black & Harper, 1979). However, as has been found in previous studies (Boom & Mori, 1982; Putz, 1980), we found that neither tree bark type nor buttress presence significantly influenced the probability of hosting a liana.

### 4.4 Prediction of future liana impacts upon fragmented forests

It is clear that multiple environmental and ecological determinants influence liana infestation of trees (Hegarty, 1991; van der Heijden et al., 2008; Putz, 1980, 1984a, 1984b; Schnitzer & Bongers, 2002) and that these determinants likely interact synergistically (van der Heijden et al., 2008; Laurance et al., 2014a; Sfair et al., 2016). Further, attributes of the liana community (abundance, size distribution class, and climbing guild) all respond to these influences. Nevertheless, liana abundance alone is often used as a proxy to infer likely liana impact (and future impact) on fragmented forests (e.g., Campbell et al., 2015a, 2015b; Schnitzer, Bongers, & Wright, 2011; Wright, 2010). However, our findings identified liana size as a possible indicator of potential liana infestation rates of trees and future liana impact. Lianas are known to significantly impact forest community processes such as decreasing forest carbon storage capacity (Schnitzer et al., 2014; van der Heijden et al., 2013; van der Heijden, Phillips, & Schnitzer, 2015a; van der Heijden, Powers, & Schnitzer, 2015b), arresting forest succession (Paul & Yavitt, 2011; Schnitzer & Bongers, 2005; Schnitzer & Carson, 2010; Tymen et al., 2015), and causing differential mortality between host species (Clark & Clark, 1990; Schnitzer & Bongers, 2002). However, the contribution to these impacts made by large lianas is often not determined. And, as above, most focus is on liana abundance. Consequently, when assessing tropical closed‐canopy forests for liana impacts and determining future management strategies, as well as the clearly justifiable assessment of overall liana abundance, considerable useful information may be attained through the assessment of the liana size (DBH) frequency distributions at sites.

## 5 CONCLUSION

Forest fragmentation significantly alters the abundance and community composition of lianas and their ecological relationships with trees. Liana abundance increased significantly within fragmented forests in response to the increased disturbance of fragmented forest edges. However, liana infestation rates of trees were not significantly different between fragmented and intact forests but was influenced by liana abundance and average liana size (DBH). Abundance and size distribution responded in opposing ways to environmental drivers, potentially explaining the finding of no significant difference in infestation rates of trees existing in fragmented and intact forests. Moreover, the increased disturbance of forest edges resulted in a shift in the composition of liana climbing guilds, likely due to a change in the size of available climbing trellises. Finally, our findings clearly identify the fact that effective control of lianas in forest fragments requires management practices which directly focus on minimizing forest edge disturbance.

## CONFLICT OF INTEREST

None declared.

## AUTHOR CONTRIBUTION

M.J.C., W.E., and W.F. L. conceived the idea and designed the project. M.J.C. and A.M. collected the data. M.J.C., W.E., A.M., and G.P. analyzed the data. M.J.C. drafted the article, and all authors (including D.M.) contributed to critical revisions of the manuscript.
