# Supplementary material for: Edge disturbance drives liana abundance increase and alteration of liana–host tree interactions in tropical forest fragments
Source: Ecol Evol. 2018 Apr 2;8(8):4237–51. doi: 10.1002/ece3.3959 (PMC5916267; doi:10.1002/ece3.3959)
Supplement: Supplementary file 1 [file ECE3-8-4237-s001.docx]

## Supporting Information

Table S1. Generalized linear mixed model (Poisson with log link) for the impact of forest fragmentation effects and environmental characteristics on tree abundance. Only the significant explanatory variables are shown. Forest edge distance = mid-distance of plot to the forest edge. All explanatory variables were standardized prior to the analysis ((x - mean(x)) / SD(x)).

|  | Estimate | SE | Z value | P |
| --- | --- | --- | --- | --- |
| Intercept | 3.499 | 0.037 | 93.60 | **< 0.001** |
| Forest edge distance | -0.092 | 0.032 | -2.81 | **0.004** |
| Liana abundance | 0.044 | 0.030 | 1.45 | 0.147 |
| Tree DBH | -0.053 | 0.032 | -1.66 | 0.096 |
| Plot forest carbon | 0.090 | 0.028 | 3.15 | **0.001** |
| Altitude | -0.088 | 0.027 | -3.24 | **0.001** |
| Forest Type (Fragmented) | -0.215 | 0.058 | -3.71 | **< 0.001** |

Table S2. Generalized linear mixed model (gamma with log link) for the impact of forest fragmentation effects and environmental characteristics on tree diameter breast height (DBH). Only the significant explanatory variables are shown. All explanatory variables were standardized prior to the analysis ((x - mean(x)) / SD(x)).

|  | Estimate | SE | t value | P |
| --- | --- | --- | --- | --- |
| Intercept | 2.863 | 0.025 | 110.22 | **< 0.001** |
| Liana DBH | 0.068 | 0.017 | 3.95 | **< 0.001** |
| Plot forest carbon | 0.043 | 0.017 | 2.52 | **0.011** |
| Altitude | 0.068 | 0.019 | 3.59 | **< 0.001** |
| *Calamus* spp. relative abundance | 0.038 | 0.020 | 1.92 | 0.054 |
| Canopy cover | 0.038 | 0.018 | 2.08 | **0.037** |
| Forest Type (Fragmented) | 0.127 | 0.040 | 3.17 | **0.001** |

Table S3. Generalized linear mixed model (gamma log link) for the impact of forest fragmentation effects and environmental characteristics on logit transformed proportional canopy cover. Only the significant explanatory variables are shown. Forest edge distance = mid-distance of plot to the forest edge (m) and this was analyzed using a quadratic term (x1 +x12) to enable better model fit. All explanatory variables were standardized prior to the analysis ((x - mean(x)) / SD(x)).

|  | Estimate | SE | t value | P |
| --- | --- | --- | --- | --- |
| Intercept | 1.606 | 0.039 | 41.09 | **< 0.001** |
| Forest edge distance | 0.237 | 0.030 | 7.79 | **< 0.001** |
| Quadratic term forest edge distance (x_1_ +x_1_^2^) | -0.142 | 0.028 | -5.10 | **< 0.001** |
| Forest type (Fragmented) | -0.172 | 0.045 | -3.75 | **< 0.001** |
| Proportionate liana infestation of trees | 0.046 | 0.023 | 1.94 | 0.051 |
| Altitude | -0.064 | 0.020 | -3.11 | **0.001** |
| Forest edge distance : Forest type interaction | -0.127 | 0.042 | -3.03 | **0.002** |
